# The Ral Exchange Factor Rgl2 Promotes Cardiomyocyte Survival and Inhibits Cardiac Fibrosis

**DOI:** 10.1371/journal.pone.0073599

**Published:** 2013-09-17

**Authors:** Rebecca L. Scotland, Leah Allen, Leah J. Hennings, Ginell R. Post, Steven R. Post

**Affiliations:** 1 Department of Molecular and Biomedical Pharmacology, University of Kentucky, Lexington, Kentucky, United States of America; 2 Department of Pharmaceutical Sciences, University of Kentucky, Lexington, Kentucky, United States of America; 3 Department of Pathology, University of Arkansas for Medical Sciences, Little Rock, Arkansas, United States of America; University of Illinois at Chicago, United States of America

## Abstract

Cardiomyocytes compensate to acute cardiac stress by increasing in size and contractile function. However, prolonged stress leads to a decompensated response characterized by cardiomyocyte death, tissue fibrosis and loss of cardiac function. Identifying approaches to inhibit this transition to a decompensated response may reveal important targets for treating heart failure. The Ral guanine nucleotide disassociation (RalGDS) proteins are Ras-interacting proteins that are upregulated by hypertrophic stimuli. The Ral guanine nucleotide dissociation stimulator-like 2 (Rgl2) is a member of the RalGDS family that modulates expression of hypertrophic genes in cardiomyocytes. However, the pathophysiologic consequence of increased Rgl2 expression in cardiomyoctyes remains unclear. To evaluate the effect of increasing Rgl2 activity in the heart, transgenic mice with cardiac-targeted over-expression of Rgl2 were generated. Although Ral activation was increased, there were no apparent morphologic or histological differences between the hearts of Rgl2 transgenic and nontransgenic mice indicating that increased Rgl2 expression had no effect on basal cardiac phenotype. To determine if Rgl2 modulates the cardiac response to stress, mice were infused with the ß-adrenergic receptor agonist, isoproterenol. Isoproterenol infusion increased heart mass in both Rgl2 transgenic and nontransgenic mice. However, unlike nontransgenic mice, Rgl2 transgenic mice showed no morphologic evidence of cardiomyocyte damage or increased cardiac fibrosis following isoproterenol infusion. Increased Rgl2 expression in cultured cardiomyocytes stimulated Ral activation and inhibited staurosporine-induced apoptosis via increased activation of PI3-kinase. Activation of the PI3-kinase signaling pathway was confirmed in hearts isolated from Rgl2 transgenic mice. Increased expression and function of Rgl2 in cardiomyocytes promotes activation of the PI3-kinase signaling cascade and protects from carciomyocyte death and pathologic cardiac fibrosis. Taken further, these results suggest that Rgl2 upregulation in hypertrophic hearts may be a protetive mechanism, and that Rgl2 may be a novel therapeutic target in treating heart disease.

## Introduction

In response to stress, the heart maintains cardiac output through a compensatory response that includes expression of fetal cardiac genes, increased cardiomyocyte size and enhanced contractile force (reviewed in [1], [2], [3]). Prolonged cardiac stress can lead to cardiomyocyte death, cardiac fibrosis and a progressive loss of cardiac function [4], [5], [6]. Inhibiting the transition from a compensated to decompensated cardiac phenotype is key to understanding and treating heart failure.

The monomeric GTP-binding protein Ras is a key regulator of cell growth and function. Incubation of cardiomyocytes with agonists that transiently activate Ras (e.g., insulin, phenylephrine) induces cardiomyocyte growth and survival [7], [8], [9]. However, in mice with cardiac targeted expression of a constitutively active Ras (V12Ras), chronic Ras activation promotes cardiomyocyte hypertrophy, induction of hypertrophic genes and early lethal heart failure [10], [11]. Ras mediates its effects by interacting with several effectors including Raf, PI3-kinase (PI3K), and Ral-GDS proteins. In contrast to Raf and PI3K, which mediate cardiac hypertrophy with preserved contractile activity and function [8], [12], [13], [14], the potential role of Ral-GDS proteins in cardiac hypertrophy is not well defined.

The RalGDS family includes Ral-GDS, Rgl, Rgl2 and Rgl3 (reviewed in [15]). Rgl2, also referred to as the Ral guanine nucleotide dissociation stimulator-like 2 (Rlf), was identified in a yeast two hybrid screen of a cardiac cDNA library as a Ras-interacting protein in the heart [16]. In neonatal rat ventricular myocytes (NRVMs), expression of Rgl2 transactivated the atrial natriuretic peptide and myosin light chain promoters, and potentiated phenylephrine-mediated gene expression [16]. These results indicate that Rgl2 is a novel regulator of transcriptional responses in cardiomyocytes. A role for Ral-GDS and Ral activation in cardiomyocyte hypertrophy is further supported by the findings that hypertrophic agents increased RalGDS expression in cardiomyocytes, expression of constitutively-active Ral induced cardiomyocyte hypertrophy and Ral activity is increased in hypertrophied hearts [17].

The aim of this study was to investigate the effect of increased expression of the Ral-GDS family member, Rgl2, in the hearts of transgenic mice and isolated cardiomyocytes. Results obtained using both transgenic mice with cardiac-targeted Rgl2 expression (Rgl2-Tg) and adenoviral-mediated expression of Rgl2 in cultured cardiomyocytes identify a novel cardioprotective effect of Rgl2 mediated by activation of the PI3K/Akt signaling pathway.

## Materials and Methods

### Ethics Statement

All animal studies were carried out under the approval and supervision of the Division of Laboratory Animal Research Committees of the University of Kentucky (protocol #00670) and the University of Arkansas for Medical Sciences (protocol #3225), in accordance with the National Institutes of Health (NIH) Guidelines for the Care and Use of Laboratory Animals.

### Rgl2 Transgenic Mice

cDNA for the mouse ortholog of Rgl2 (i.e., Rlf) with a hemagglutinin epitope (HA) tag was provided by Dr. Rob Wolthuis (The Netherlands Cancer Institute, Amsterdam). This sequence was subcloned into the mouse α-myosin heavy chain (α-MyHC) promoter (provided by Dr. Jeffery Robbins, University of Cincinnati) and used to generate transgenic mice (Rgl2-Tg) at the University of Kentucky transgenic core facility. Genomic DNA was isolated from founder mice and shown to express the expected 241 base pair DNA using primers specific for the α-MyHC promoter and Rgl2 insert (forward, 5′-ctg gtc aga cac ctc ttg ga-3′; reverse, 5′-cct tga cct cag agc caa aa-3′). Founders were bred with Black Swiss mice and progeny screened for transgene transmission via PCR. Two transgenic mouse lines, one of which was bred to homozygosity, were used and yielded similar results. Results obtained using mice that are homozygous for the Rgl2 transgene are presented.

### Generation of Rgl2 Antibody

A monoclonal antibody specific to wild-type Rgl2 was generated in collaboration with BD Biosciences. Subsequently, a commercially available Rgl2 antibody (Abcam) was used with identical results.

### Isoproterenol Infusion of Mice

Two-month-old male transgenic and non-transgenic littermates were anesthetized with an intraperitoneal injection of ketamine (0.0065 mg/g) and xylazine (0.013 mg/g). Mini-osmotic pumps (model 2002, Alzet®) filled with either 0.02% ascorbic acid (AA, vehicle control) or isoproterenol (Sigma Chemical) were implanted subcutaneously via a small mid-scapular incision. The concentration of isoproterenol was calculated to deliver an average of 30 mg/kg/day during the infusion period. After 14 days of continuous infusion, the mice were anesthetized, hearts rapidly excised rinsed in ice cold saline, and then processed for histological or biochemical analysis.

For histological examination, excised hearts were bisected, fixed with 10% buffered formalin, embedded in paraffin, and sectioned at 4 µm thicknesses. Sections were stained with hematoxylin and eosin (H&E) for morphologic evaluation, modified elastic trichrome for evaluation of collagen, or incubated with an anti-HA rabbit polyclonal antibody (Upstate, Lake Placid, NY) followed by a peroxidase-conjugated secondary antibody for immunohistochemical detection of Rgl2. Images of trichrome stained slides were digitally captured using an Aperio® Scanscope (Aperio, Vista, CA) and area of fibrosis quantified using the Aperio color deconvolution algorithm. H&E-stained slides were examined and myocyte vacuolation was graded on a scale of 0 (no vacuolization) to 4 (severe vacuolization) by a veterinary pathologist who was blinded to treatment and genotype. Cross-sectional area of myocytes was determined using Image Pro Plus software (Media Cybernetics).

For biochemical analysis, cardiac tissue homogenates were prepared by rapidly freezing ventricular tissue in liquid nitrogen. A hammer pre-chilled in liquid nitrogen was used to pulverize ventricles into a fine powder. The powdered hearts were suspended in 1 mL of MBST/OG buffer [25 mM MES, 150 mM NaCl, 60 mM octyglucopyranoside, 1% Triton-X100, pH 6.4, and protease/phosphatase inhibitors (Pierce)], homogenized with an ultrasonic homogenizer, and incubated on ice for 30 minutes. Lysates were cleared of non-soluble material by centrifugation at 300×g for 3 minutes at 4°C and the protein concentration determined using the DC Lowry protein assay (BioRad) with BSA as a standard. Subsequently, equal amounts of protein were resolved by 12% SDS-PAGE. Western blot analysis was performed using antibodies to: Akt; phospho^Ser473^-Akt; the active (cleaved) and full-length (procaspase-3) forms of caspase-3; rabbit polyclonal Bax antibody, or rabbit polyclonal Bcl-xL antibody (Cell Signaling, Danvers, MA), and the appropriate species-specific horseradish peroxidase (HRP)-conjugated secondary antibody (Cell Signaling). Immunoreactive bands were visualized using enhanced Super Signal West Pico chemiluminesence (Pierce Biotechnology) and band intensity quantified using a Kodak Image Station.

### Generation of Adenoviral Vectors

HA tagged-Rgl2 cDNA was subcloned into an adenoviral backbone plasmid, AdEasy-1 (Stratagene) and recombinant adenoviral plasmids transfected into HEK293 cells. The resulting viral particles (AdRgl2) were purified by gradient centrifugation and viral infectivity (ifu/ml) determined (Biogenesis Adenovirus). Adenovirus lacking the Rgl2 insert (AdNull) was provided by Dr. Nancy Webb (University of Kentucky, Lexington, KY).

### Isolation and Culture of Cardiomyocytes

Neonatal Rat Ventricular Myocytes (NRVMs) were isolated from 1–2 day old Sprague-Dawley rats according to a previously described protocol with modifications [18]. Briefly, hearts were excised and enzymatically digested at 37°C for 2 hours with collagenase type II (Worthington) and pancreatin (Sigma). Following tissue digestion, the isolated cells were incubated in an uncoated flask for 2 hours to allow for the attachment of non-myocyte cells. The subsequent cardiomyocyte-enriched suspension was plated at a density of 0.3×10^6^ to 0.5×10^6^ cells/ml on gelatin-coated plates and maintained at 37°C under 5% CO_2_ in DMEM/Medium 199 containing 10% equine serum, 5% fetal bovine serum (FBS), and penicillin/streptomycin (Invitrogen). Cardiomyocytes were allowed to attach for 24 hours, washed, and maintained in culture for an additional 24 hours prior to adenoviral infection.

HL-1 cardiomyocytes were provided by Dr. William Claycomb (Louisiana State University Medical Center, New Orleans, LA) and maintained in culture as previously described [19]. HL-1 cardiomyocytes were grown to 1.0–1.5×10^6^ cells/well in 6-well culture plates prior to adenoviral infection.

### Cardiomyocyte Infection and Treatment

Cardiomyocytes (NRVMs or HL-1) were incubated with AdRgl2 or AdNull virus (90 ifu/cell) in serum-free media. After 2 hours, FBS was added to the media at a final concentration of 1% (NRVMs) or 5% (HL-1s) and the incubation continued overnight. Infection of either NRVMs or HL-1 cardiomyocytes with 90 ifu/cell resulted in an infection efficiency of ∼80% without affecting cell viability, as assessed by trypan blue exclusion (data not shown). Cardiomyocytes were then washed and maintained in media without serum for 2 hours prior to treatment with wortmannin (200 nM; Biomol), staurosporine (1 µM; Biomol), or the appropriate vehicle for the indicated time prior to cell lysis.

Following infection and treatment with pharmacological agents or vehicle, cardiomyocytes were lysed for 30 minutes on ice in MBST/OG buffer. The protein concentration of each supernatant was determined using the DC Lowry protein assay and equal amounts of lysate protein resolved by 12% SDS-PAGE. For NRVM samples treated with staurosporine, cardiomyocytes were lysed directly in Laemmli sample buffer and resolved by 4–12% gradient SDS-PAGE. Western blot analysis was performed using antibodies to: Akt; phospho^Ser473^-Akt; the active (cleaved) and full-length (procaspase-3) forms of caspase-3; and PARP (Cell Signaling) and the appropriate species-specific horseradish peroxidase (HRP)-conjugated secondary antibody (Cell Signaling). Immunoreactive bands were visualized using enhanced Super Signal West Pico chemiluminesence (Pierce Biotechnology) and band intensity quantified using a Kodak Image Station. The intensity of immunoreactive bands was normalized to a control protein as indicated in the figure legends.

### Ral Activation Assay

To assess Ral activation in cardiac tissue and cardiomyocytes, homogenates or cardiomyocytes (lysed in buffer containing 25 mM Tris-HCL, pH 7.5, 40 mM NaCl, 30 mM MgCl_2_, 1% Ipegal, 1 mM DTT, and protease/phosphatase inhibitors) were incubated with a GST fusion protein which specifically binds to the active, GTP-bound form of Ral (GST-RalBD; provided by Dr. Doug Andres, University of Kentucky, Lexington, KY) as previously described [16], [20]. Briefly, ventricular homogenates or cardiomyocyte lysates (500 µg) were incubated with GST-RalBD bound agarose beads for 1 hour at 4°C with gentle rotation. The beads were washed, proteins resolved by 12% SDS-PAGE, and Ral detected using a RalA-specific antibody (BD Bioscience). The relative amount of active Ral (Ral-GTP) was determined by normalizing to total Ral detected in the lysate.

### Statistical Analysis

Experiments performed with Rgl2 transgenic mice were repeated in two lineages with similar results. Results are reported as mean ± standard error (SEM). A significant difference between treatment groups was determined by t-test for individual comparisons or for multiple comparisons by one-way ANOVA and the post-hoc test indicated in legends. All analyses were completed using GraphPad Prism software. Statistical significance was accepted for p<0.05.

## Results

**Figure 1:**
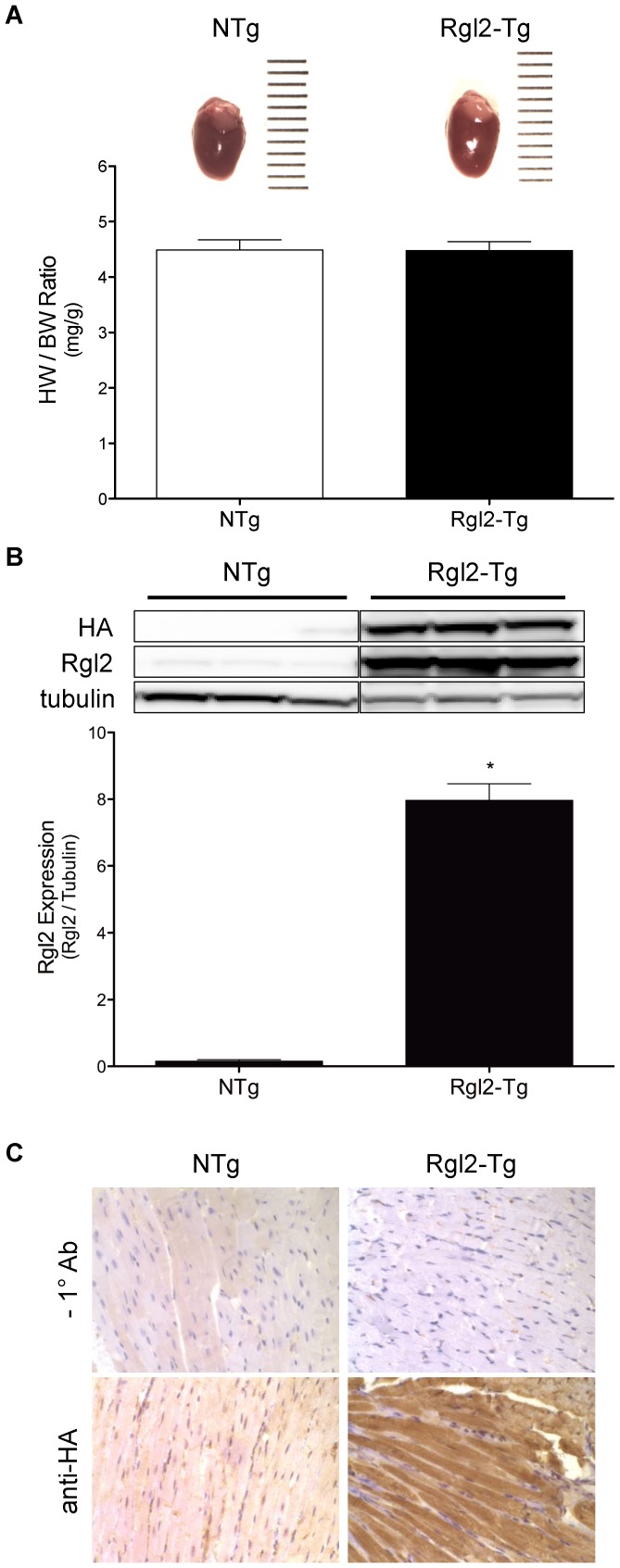
Transgenic expression of Rgl2 in mice. *A*) Representative pictures of hearts from nontransgenic (NTg) and transgenic mice with cardiac specific overexpression of Rgl2 (Rgl2-Tg). Ruler gradations are 1 mm. Graph represents the mean ± SEM from at least 4 different aged-matched hearts. * denotes significant difference from NTg. *B*) Tissue homogenates from hearts of NTg and Rgl2-Tg mice were probed with an antibodies to the hemagglutinen (HA) epitope and Rgl2. Shown are a representative blot from a single experiment and the mean ± SEM (n = 3) of Rgl2 expression normalized to tubulin. * denotes significant difference from NTg. *C*) Photomicrographs of histological sections of ventricular tissue demonstrating Rgl2 expression in myofibrils of Rgl2-Tg. The sections depicted in the left panels were incubated without primary antibody and the right sections were incubated with a polyclonal antibody to the HA epitope (200×magnification).

Rgl2 was identified as a Ras interacting protein in the human heart that potentiated transcriptional activation of hypertrophic genes in cultured NRVMs [16]. To correlate these previous findings with the in vivo effects of Rgl2, the α-MyHC promoter was used to express the cDNA for HA-tagged Rgl2 in the hearts of transgenic mice. The hearts of Rgl2 transgenic (Rgl2-Tg) and nontransgenic (NTg) mice showed no gross morphologic differences at 2 months of age (**Figure 1A**) or at 12 months (data not shown). Western blot analysis using a Rgl2-specific antibody (**Figure 1B**) showed increased Rgl2 protein expression in cardiac tissue of Rgl2-Tg mice, confirming transgene expression. Immunohistochemical analysis of ventricular tissue using an antibody to the HA-epitope verified transgenic protein (HA-Rgl2) expression specifically in cardiac myofibrils of the Rgl2-Tg mice (**Figure 1C**).

**Figure 2:**
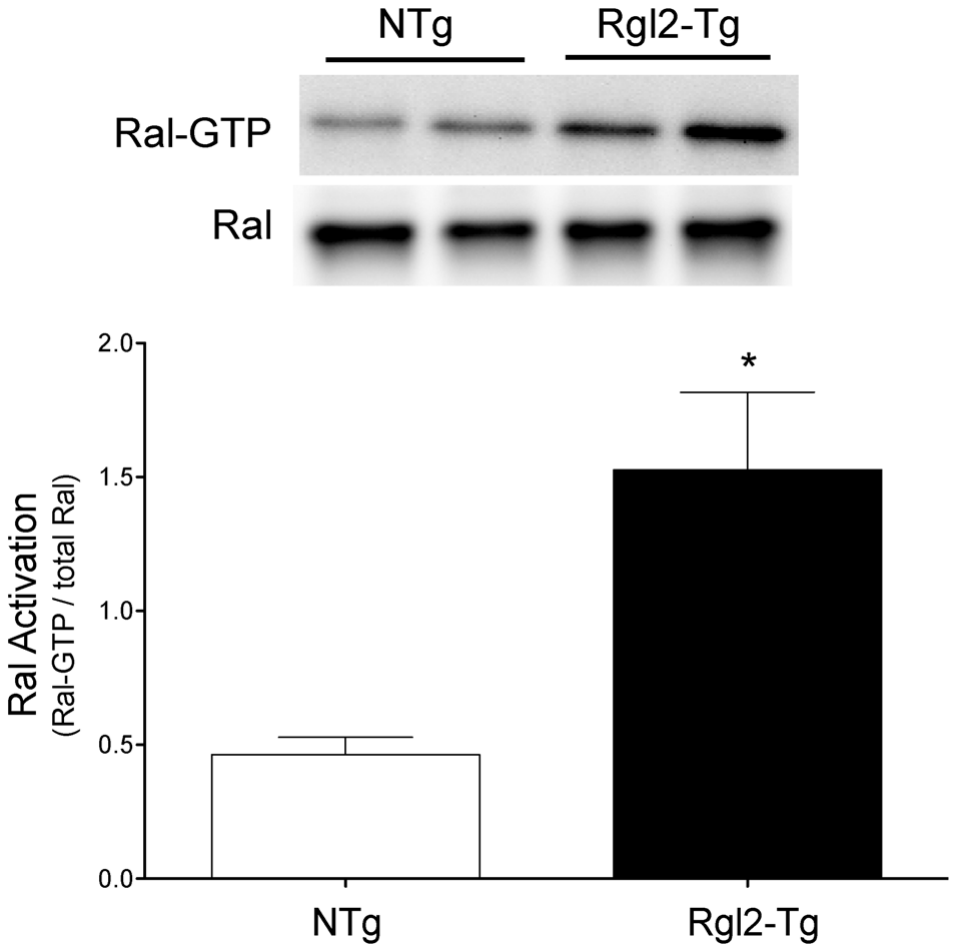
Expression of Rgl2 activates Ral. Heart homogenates from Rgl2-Tg and NTg littermate mice were prepared and the amount of GTP-bound Ral (active) assessed by immunoblotting. Total Ral was assessed on separate immunoblots. Total and GTP-bound Ral were quantified and a representative image from a single experiment and the means ± SEM from at least 6 individual mice are shown. * denotes significant difference from NTg.

Rgl2 promotes the exchange of GTP for GDP on the monomeric G protein Ral. Therefore, to verify functionality of the expressed Rgl2 protein, Ral-GTP levels in the hearts of Rgl2-Tg and NTg mice were quantified using an affinity isolation assay for activated Ral [16], [20]. GTP-bound active Ral was isolated from ventricular homogenates using an immobilized GST-Ral binding-domain fusion protein, which only associates with GTP-bound (i.e., active) Ral protein. The isolated proteins were immunoblotted with Ral-specific antibody to quantify the amount of activated Ral protein. Compared to controls, Ral activation was increased (2–3-fold) in the hearts of Rgl2-Tg mice (**Figure 2**). Western blots of cardiac homogenates indicated that Rgl2-mediated increases in Ral-GTP were not associated with increased Ral protein levels.

**Figure 3:**
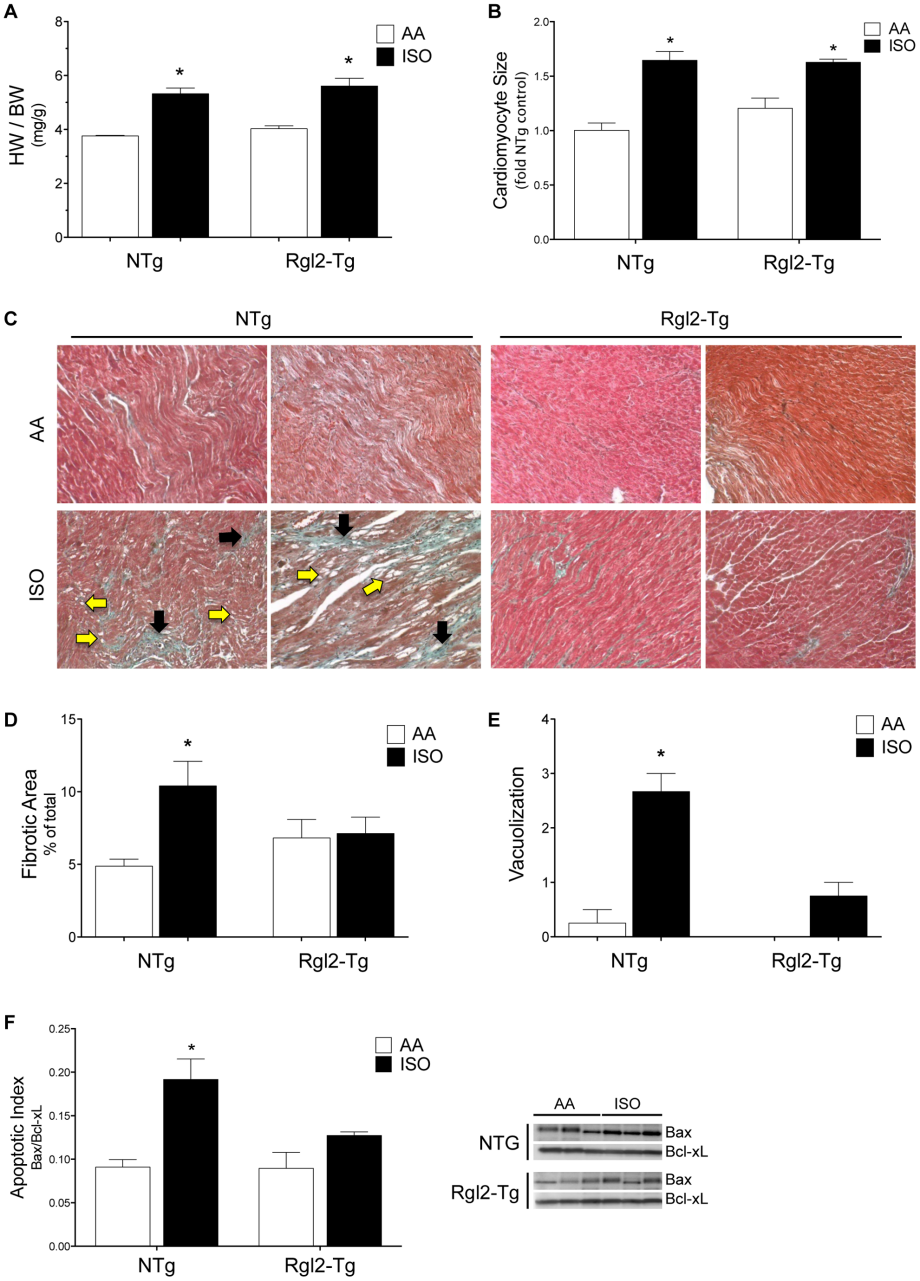
Effect of Rgl2 on isoproterenol induced cardiac hypertrophy. Rgl2-Tg and NTg littermates were continuously infused with isoproterenol (30 mg/kg/day; ISO) or vehicle (ascorbic acid; AA) for 14 days via mini-osmotic pumps. The effect of isoproterenol infusion on cardiac hypertrophy (*A,B*) and pathology (*C–E*) were determined. *A*) Heart weight (HW) and body weight (BW) were determined and are expressed as the heart weight (HW) to body weight (BW) ratio. *B*) Cardiomyocyte cross-sectional area was quantified by morphometric analysis of individual cells and expressed relative to untreated NTG mice. *C*) Pathological changes in hearts of Rgl2-Tg and NTg mice following infusion were assessed using a modified elastic trichrome stain to visualize collagen (green). Shown are representative sections from two different hearts from NTg and Rgl2-Tg mice at 200×magnification. Representative areas of fibrosis are indicated by black arrows, and myocyte vacuolization by yellow arrows. Fibrotic area (D) and extent of vacuolization (E) were quantified by morphometric analysis. Results are expressed as the mean ± SEM obtained from at least 9 different mice. *F*) Apoptotic index was determined by western blotting for Bax and Bcl-xl in left ventricular homogenates and expressed as the Bax/Bcl-xl ratio. A representative blot of lysates from 3 mice of each treatment and graph of mean ± SEM from 9 mice are shown. * denotes significant difference from AA infused NTg mice using Dunnett’s multiple comparison test.

The results presented above suggest that increased expression and function of Rgl2 alone does not alter cardiac morphology. To determine if increasing Rgl2 expression alters the response of cardiomyocytes to increased stress, 2-month old Rgl2-Tg and NTg mice were continuously infused for 2 weeks with the ß-adrenergic receptor (ß-AR) agonist isoproterenol (ISO; 30 mg/kg/day) or vehicle (AA; ascorbic acid). Isoproterenol-infused animals are a commonly used cardiac stress model for studying cardiac hypertrophy and inducing cardiac dysfunction [21], [22], [23], [24]. Compared to vehicle infused mice, isoproterenol infusion increased the heart weight to body weight ratio and heart weight normalized to tibia length (data not shown) to the same extent in NTg and Rgl2-Tg mice (**Figure 3A**). Isoproterenol-induced increased heart sized was paralleled by an increase in cardiomyocyte cross-sectional area (**Figure 3B**). However, compared to vehicle infused mice, hearts prepared from isoproterenol-infused NTg mice displayed increased collagen deposition (black arrows) indicating increased fibrosis (**Figure 3C and D**) and cardiomyocyte vacuolization (yellow arrows), indicating increased cardiomyocyte injury (**Figure 3C and E**). Increased cardiomyocyte injury was assoicated with an increase in apoptotic index (Bax/Bcl-xl ratio) indicating an increase in cardiomyocyte apoptosis (**Figure 3F**). In contrast, Rgl2-Tg hearts displayed no significant increases in fibrosis, vacuolization, or apoptotic index following isoproterenol treatment. These data indicate that increased Rgl2 expression does not affect ß-AR-induced increases in cardiac mass; however, Rgl2 expression protects from ß-AR-induced cardiomyocyte injury, apoptosis, and increased cardiac fibrosis.

**Figure 4:**
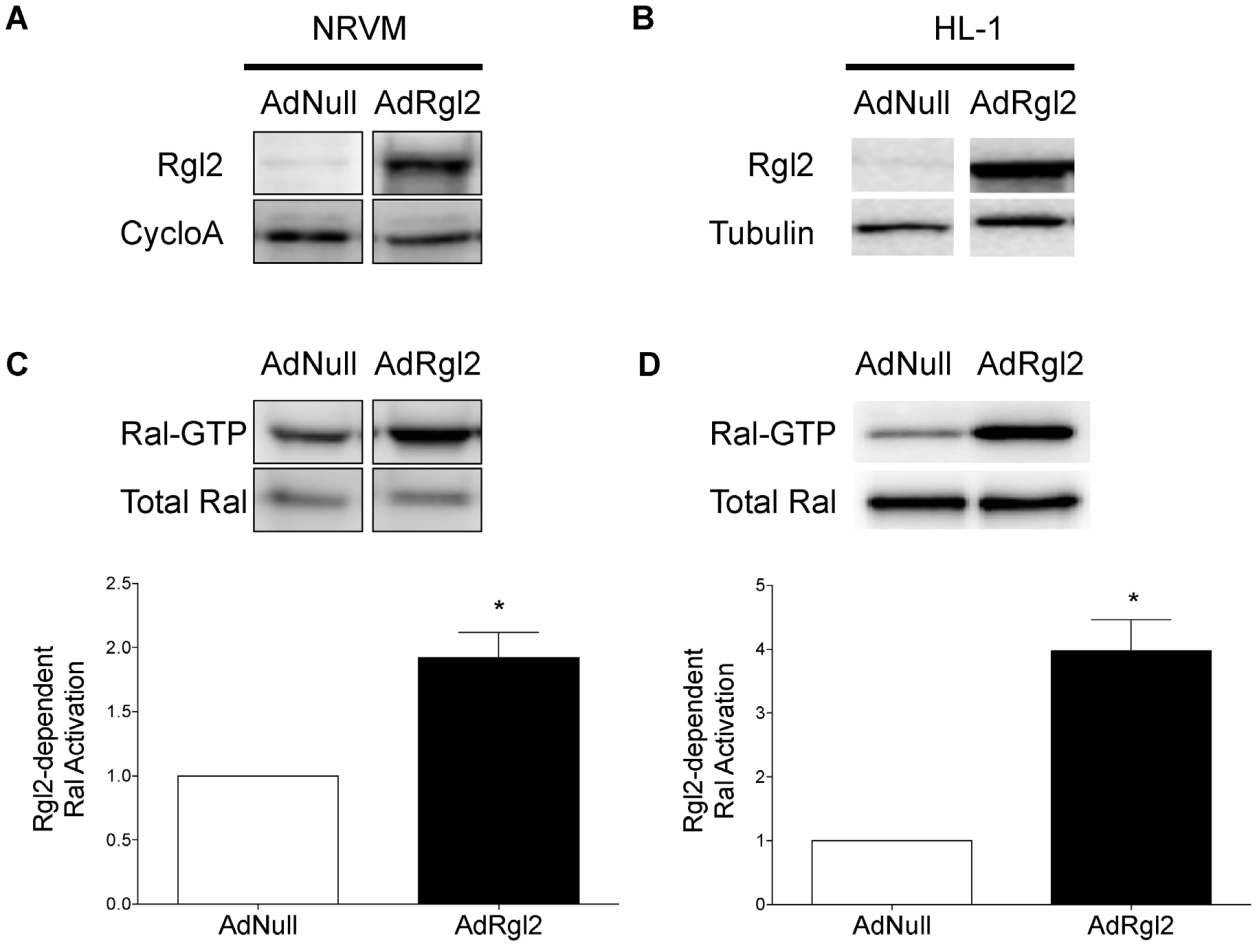
Adenoviral-mediated expression of Rgl2 in cardiomyocytes. NRVMs (A and C) or HL-1 cardiomyocytes (B and D) were infected with 90 ifu/cell of AdNull or AdRgl2. After infection, cell lysates were prepared and analyzed for Rgl2 expression (A and B) via immunoblotting using Rgl2 antibodies, and function (C and D) by assessing Ral activation. Rgl2 expression was normalized to cyclophilin A (cycloA) or tubulin. Ral activation was determined as described in Figure 2 and are presented as the ratio of values obtained in AdRgl2- to AdNull-infected cells. Shown are representative immunoblots of a single experiment and the means ± SEM of at least 4 separate experiments. * denotes significant difference from AdNull infected cells.

To assess the mechanism by which increased Rgl2 expression in cardiomyocytes protects from ß-AR-induced cardiac injury, HA-Rgl2 was expressed in cultured NRVMs and HL-1 cells, a commonly used cardiomyocyte cell line that retains the molecular and functional properties of primary adult cardiomyocytes. NRVMs and HL-1 cells were infected with a control adenovirus (AdNull) or with adenovirus encoding HA-Rgl2 (AdRgl2). Compared to AdNull-infected cells, infection of NRVMs (**Figure 4A**) or HL-1 cells (**Figure 4B**) with AdRgl2 increased Rgl2 protein expression. As shown for ventricular homogenates prepared from Rgl2-Tg mice, activated, GTP-bound Ral was significantly increased in both AdRgl2-infected NRVMs (**Figure 4C**) and HL-1 cells (**Figure 4D**) compared to AdNull-infected cardiomyocytes. There was no effect on total Ral protein expression in either cell system. These results are consistent with the previously described ability of Rgl2 to enhance guanine nucleotide exchange on Ral and confirm expression of functional Rgl2 protein in cardiomyocytes following adenoviral infection.

**Figure 5:**
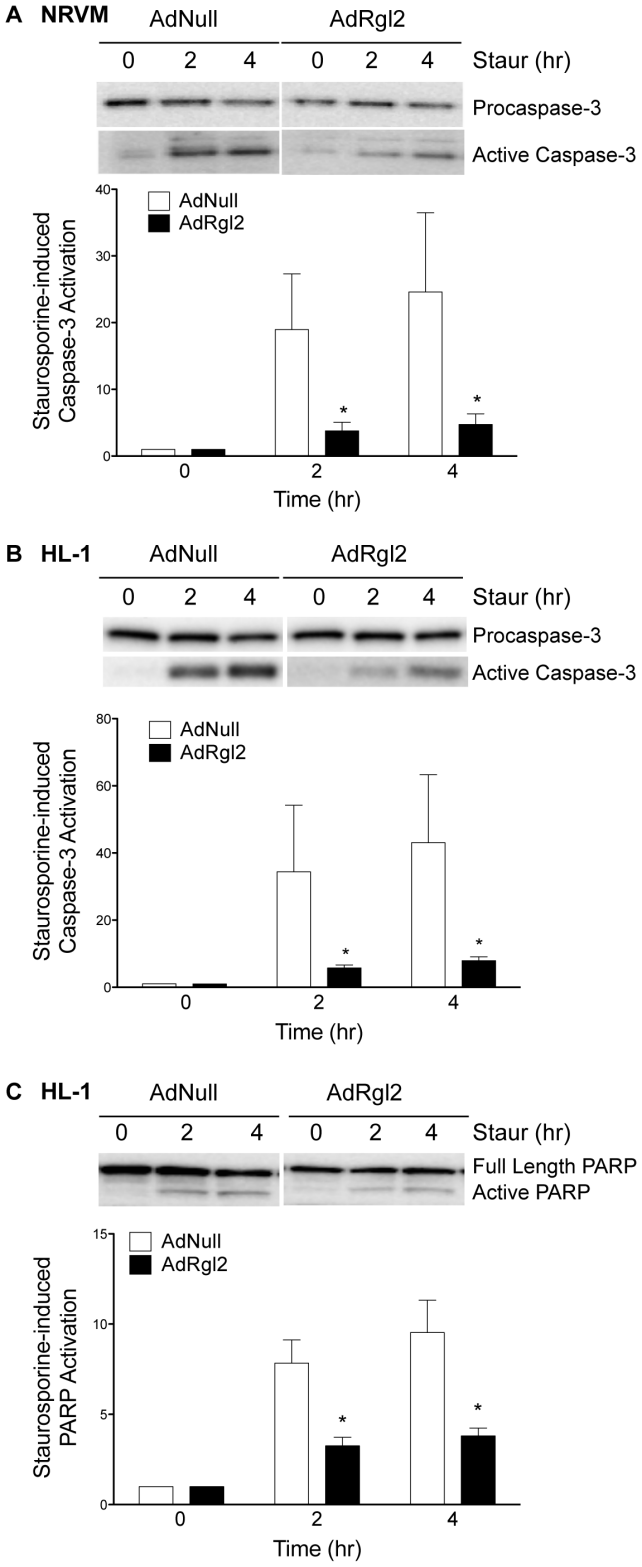
Effect of Rgl2 on apoptotic pathways in cardiomyocytes. AdNull- and AdRgl2-infected NRVMs (*A*) and HL-1s (*B* and *C*) were treated with staurosporine (Staur, 1 µM) for 0, 2, and 4 hr. Caspase-3 (*A* and *B*) and PARP (*C*) activation were determined by immunoblot of cell lysates using antibodies to activated caspase-3, procaspase-3, and PARP. Active (cleaved) caspase-3 and PARP densities were normalized to total enzyme and are expressed relative to the respective control value (0 hr Staur). Shown are representative immunoblots from a single experiment and the means ± SEM of at least 3 separate experiments. * denotes significant difference compared to AdNull infected cells.

The effect of increased Rgl2 on cardiomyocyte apoptosis was examined by incubating AdNull- or AdRgl2-infected cardiomyocytes with staurosporine, an activator of apoptotic signaling pathways [25]. Apoptosis was assessed by quantifying the active, cleaved form of caspase-3, and cleavage of its downstream effector DNAse, poly (A-ribose) polymerase (PARP). Treatment of AdNull-infected NRVMs (**Figure 5A**) and HL-1 cardiomyocytes (**Figure 5B and C**) with staurosporine for 4 hours significantly increased caspase-3 activation (**Figures 5A** and 5B) and PARP cleavage (Figure 5C). Staurosporine-induced caspase-3 activation and PARP cleavage was significantly decreased in AdRgl2-infected cardiomyocytes indicating that increasing Rgl2 expression promotes cardiomyocyte survival.

**Figure 6:**
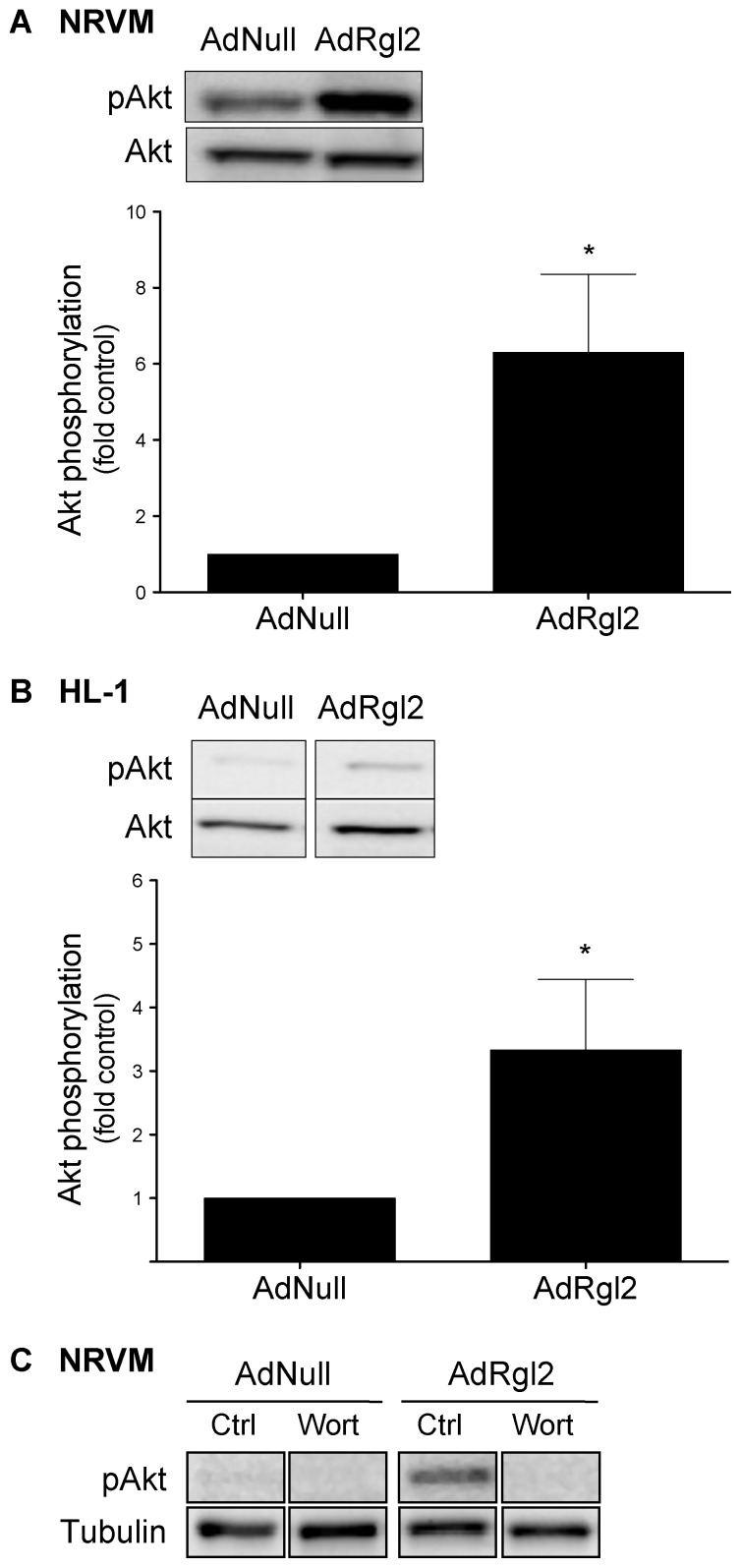
Effect of Rgl2 on the PI3K/Akt signaling pathway in cardiomyocytes. Cell lysates were collected from AdNull or AdRgl2 infected NRVMs (*A, C*) or HL-1 cardiomyocytes (*B*) and immunoblotted with antibodies for pAkt, total Akt, or tubulin. (*A* and *B*) Rgl2-induced Akt phosphorylation was quantified and normalized to AdNull infected cells. (*C*) AdNull and AdRgl2 infected NRVMs were treated with wortmannin (Wort) for 30 min prior to preparing cell lysates. Shown are representative immunoblots from a single experiment and mean ± SEM of at least 3 separate experiments. * denotes significant difference from AdNull infected cells.

Activation of the PI3K/Akt signaling pathway has been associated with increased myocardial cell survival in cell culture models and increased cardiac function in transgenic mice [7], [8], [9], [12], [13], [14], [26], [27], [28]. To determine the effect of Rgl2 on the activation PI3K/Akt signaling pathway, NRVMs were infected with AdNull or AdRgl2, and PI3K activation was assessed by immunoblotting for the active, phosphorylated form of Akt. Akt phosphorylation was significantly increased in AdRgl2-infected NRVMs (**Figure 6A**) and HL-1 cardiomyocytes (**Figure 6B**) compared to AdNull-infected controls. Furthermore, AdRgl2-induced Akt phosphorylation was inhibited by wortmannin (**Figure 6C**), confirming that Akt phosphorylation was PI3K-dependent.

**Figure 7:**
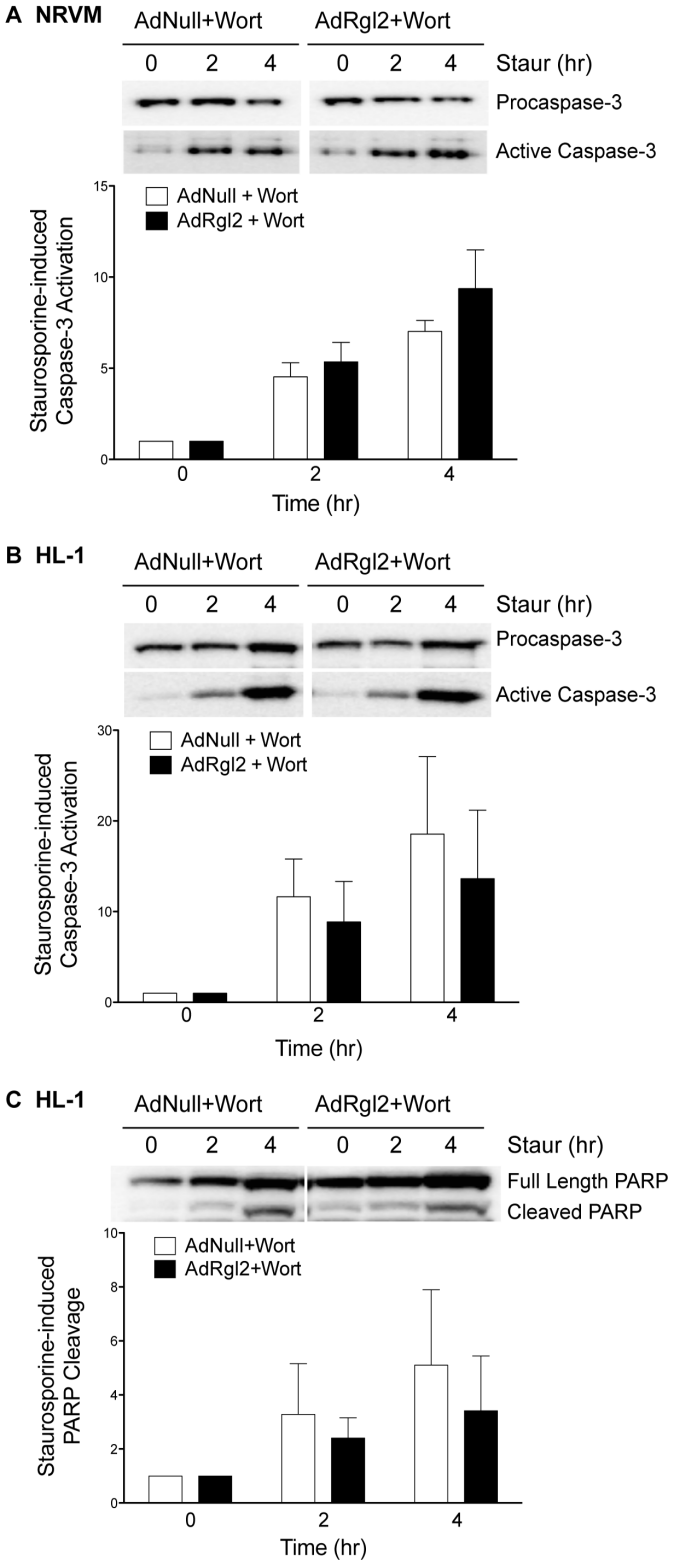
Effect of Rgl2 on staurosporine-induced caspase-3 and PARP cleavage. AdNull- and AdRgl2-infected NRVM (*A*) and HL-1 cardiomyocytes (*B* and *C*) were pretreated with vehicle or 200 nM wortmannin (Wort) for 30 min followed by exposure to 1 µM staurosporine (Staur) for 0, 2, and 4 hr. Caspase-3 (*A* and *B*) and PARP (*C*) cleavage were analyzed as described in Figure 5. Results are presented normalized to the respective baseline value (0 hr Staur). Values are expressed as mean ± SEM of at least 3 separate experiments. * denotes significant difference from AdNull infected cells.

The contribution of PI3K/Akt activation in mediating protection of cardiomyocytes from staurosporine-induced injury was assessed using the PI3K-specific inhibitor wortmannin (200 nM; Wort). Rgl2-mediated reduction in caspase-3 and PARP cleavage was abolished by pretreating NRVMs (**Figure 7A**) and HL-1 cells (**Figure 7B and C**) with wortmannin, confirming that PI3K activation is required for Rgl2-enhanced cell survival. Together, these data indicate that Rgl2 promotes cardiomyocyte survival by activating the PI3K/Akt signaling cascade.

**Figure 8:**
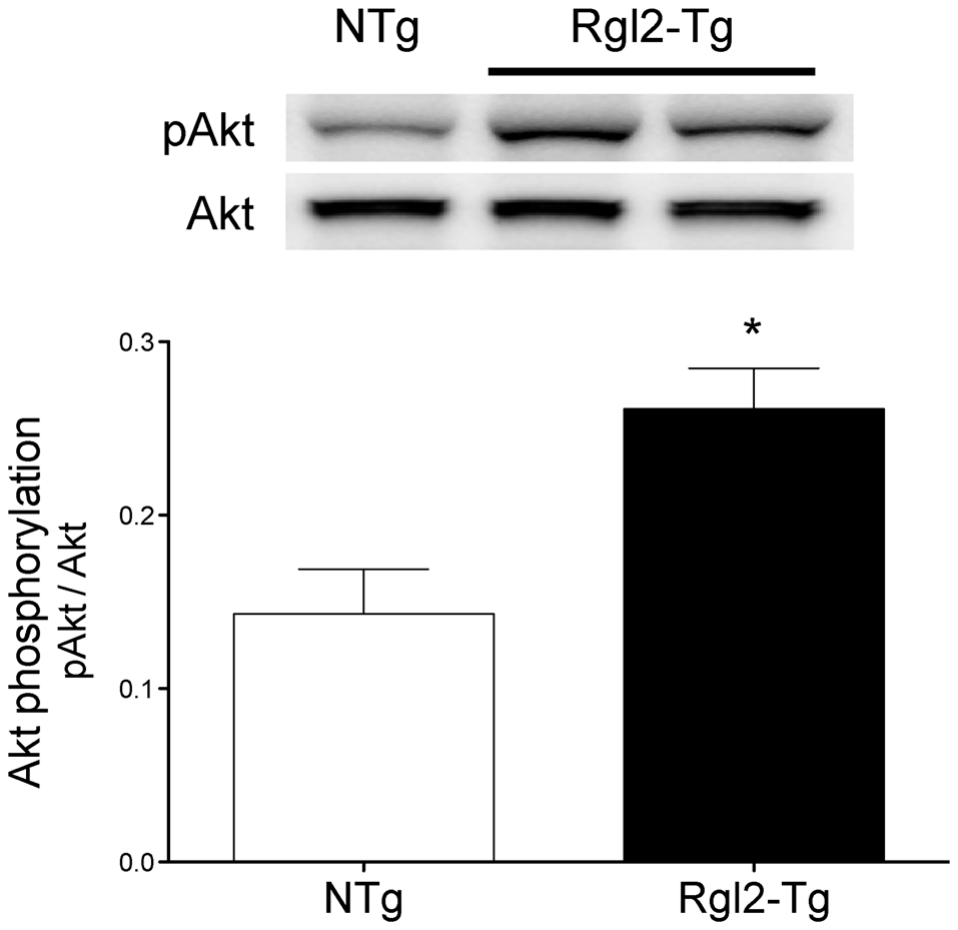
Effect of Rgl2 on the PI3K/Akt signaling pathway in transgenic mice. Heart homogenates prepared from Rgl2-Tg and NTg littermates were prepared and Akt phosphorylation quantified by immunoblotting with a phosphospecific Akt antibody and normalizing to total Akt. Shown are a representative immunoblot and the mean ± SEM of 12 different homogenates. * denotes significant difference from NTg.

Based on the findings in culture cardiomyocytes, we evaluated PI3K activation in the hearts of NTg and Rgl2-Tg mice. As shown in **Figure 8**, relative to NTg mice, there was a significant increase in pAkt detected in homogenates prepared from Rgl2-Tg mice. Thus, increasing Rgl2 expression enhanced activation of the PI3K/Akt pathway in both isolated cardiomyoctyes and in hearts of transgenic mice.

## Discussion

In this study, we examined the function of the Ras effector protein Rgl2 in the hearts of transgenic mice and in cultured cardiomyocytes. Cardiac-specific expression of constitutively active Ras initially induces cardiac hypertrophy with a rapid progression to decompensated heart failure [11]. Ral activity is also increased in hypertrophic hearts and increasing Ral activity in cardiomyocytes is sufficient to induce hypertrophy [17]. In contrast, our findings demonstrate that cardiac-targeted overexpression of Rgl2 has no apparent effect on either baseline cardiac phenotype or the increased heart mass following chronic isoproterenol infusion. Together, these findings suggest that Rgl2 does not change the basal cardiac phenotype or alter the compensatory increase in heart size resulting from stress induced by chronic ß-adrenergic receptor activation.

Cardiomyocyte injury and resultant interstitial fibrosis are important contributing factors leading to decreased cardiac function associated with multiple pathological conditions, including ischemia-reperfusion injury and heart failure [6], [29], [30]. Chronic ß-AR receptor activation induces cardiac hypertrophy, cardiomyocyte death and subsequent interstitial fibrosis [21], [22], [23]. Consistent with these reports, we found increased cardiac fibrosis, cardiomyocyte vacuolization and apoptosis in NTg mice infused with isoproterenol for two weeks. In contrast, the hearts of Rgl2-Tg mice exhibited significantly less isoproterenol-induced cardiac fibrosis, cardiomyocyte vacuolization and apoptosis indicating that increased Rgl2 activation may be cardioprotective.

To facilitate mechanistic studies of how Rgl2 mediates its cardioprotective effects, we used an adenoviral delivery approach to increase Rgl2 expression and function in primary NRVMs and in cultured HL-1 cardiomyocytes. In both cardiomyocyte model systems, infection with adenovirus encoding Rgl2 increased PI3K activity and decreased staurosporine-induced apoptosis, as assessed by decreased PARP and/or caspase-3 cleavage. Pharmacological inhibition of PI3K with wortmannin abolished Rgl2-induced Akt phosphorylation as well as Rgl2-mediated inhibition of caspase activation, demonstrating that PI3K activation mediates the observed anti-apoptotic effects of Rgl2. Modulation of the PI3K/Akt pathway in cardiomyocytes is of particular interest due to its well established role in promoting cardiomyocyte survival and function [14], [31], [32], [33], [34], [35]. For example, activation of the PI3K/Akt signaling pathway has been shown to reduce apoptosis and protect cardiac function following ischemic injury in both in vitro and in vivo models [34], [35], [36]. Our data extend these findings by demonstrating that increasing Rgl2 activity is a novel approach to induce PI3K-mediated cardiomyocyte survival. Although the increased expression of Rgl2 as achieved in this study mediates a protective effect to cardiac stress, caution must be taken in extrapolating our results to physiological conditions in which Rgl2 expression is not upregulated. Exploring the physiological roles of Rgl2 would require cardiac-targeted deletion of Rgl2.

The intermediary signaling molecules that link Rgl2 to PI3K and Akt phosphorylation in cardiomyocytes are not known. In different cell systems, RalGDS has been shown to elicit both Ral-dependent and Ral-independent effects [15], [17], [37], [38]. For example, growth factor receptor-induced phospholipase D (PLD) stimulation is mediated by a RalGDS/RalA signaling pathway in HEK293 cells [37], and increasing PLD activation has been shown to enhance PI3K/Akt signaling [39], [40], [41], [42] and mediate cardioprotective effects [43], [44]. Our results demonstrating that Rgl2 enhanced RalA activation support the possibility of a RalA-dependent mechanism for enhancing PI3K/Akt activation in cardiomyocytes. The possibility that Rgl2 has Ral independent effects in cardiomyocytes is supported by the previous finding that increased Ral activity is sufficient to induce cardiomyocyte hypertrophy [17] however, we saw no evidence that enhancing Rgl2 expression induced cardiac hypertrophy. Additional evidence for RalA-independent effects of Ral-GDS proteins include the finding that some, but not all, RalGDS-induced signals can be inhibited by a dominant-negative Ral [45], and that RalGDS directly interacts with JIP1 and PKD to induce Akt phosphorylation [38]. Similar to our results, cardiac-specific Rho overexpression was recently shown to protect from ischemia/reperfusion injury via activation of PKD [46]. Although our results do not exclude a role for PKD, a similar direct interaction between Rgl2 and PKD is unlikely to explain the current results as Rgl2-dependent Akt phosphorylation was inhibited by wortmannin indicating the requirement for PI3K activation.

## Conclusions

Overall, our results demonstrate the ability of Rgl2 to activate the PI3K/Akt signaling pathway and enhance cardiomyocyte survival without altering basal cardiac phenotype or the compensatory increase in cardiac mass following chronic ß-AR stimulation. Future studies will examine the contribution of RalA-dependent and RalA-independent signaling pathways in Rgl2-induced activation of the PI3K/Akt signaling cascade, and whether Rgl2 represents a novel therapeutic target in cardiac disease.
